# Association of IL-4RA single nucleotide polymorphisms, HLA-DR and HLA-DQ in children with *Alternaria*-sensitive moderate-severe asthma

**DOI:** 10.1186/1476-7961-8-5

**Published:** 2010-03-18

**Authors:** Alan P Knutsen, Hari M Vijay, Barbara Kariuki, Luis A Santiago, Ralph Graff, Jonathan D Wofford, Maulik R Shah

**Affiliations:** 1 Department of Pediatrics, Saint Louis University, St Louis, Missouri, 63104, USA; 2 Department of Surgery, (HLA Laboratory) Saint Louis University, St Louis, Missouri, 63104, USA; 3 Divisions of Allergy & Immunology, Saint Louis University, St Louis, Missouri, 63104, USA; 4 Department of Genetics, Saint Louis University, St Louis, Missouri, 63104, USA; 5 Health Canada, Healthy Environments and Consumer Safety Branch, Hazard Identification Division, Ottawa, ON, K1A 0K9, Canada

## Abstract

**Background:**

Asthma afflicts 6% to 8% of the United States population, and severe asthma represents approximately 10% of asthmatic patients. Several epidemiologic studies in the United States and Europe have linked *Alternaria *sensitivity to both persistence and severity of asthma. In order to begin to understand genetic risk factors underlying *Alternaria *sensitivity and asthma, in these studies we examined T cell responses to *Alternaria *antigens, HLA Class II restriction and HLA-DQ protection in children with severe asthma.

**Methods:**

Sixty children with *Alternaria*-sensitive moderate-severe asthma were compared to 49 children with *Alternaria*-sensitive mild asthma. We examined HLA-DR and HLA-DQ frequencies in *Alternaria*-sensitive asthmatic by HLA typing. To determine ratios of Th1/Th2 *Alternaria*-specific T-cells, cultures were stimulated in media alone, *Alternaria alternata *extract and Alt a1. Sensitivity to IL-4 stimulation was measured by up-regulation of CD23 on B cells.

**Results:**

Children with *Alternaria*-sensitive moderate-severe asthma trended to have increased sensitivities to *Cladosporium *(46% versus 35%), to *Aspergillus *(43% versus 28%), and significantly increased sensitivities to trees (78% versus 57%) and to weeds (68% versus 48%). The IL-4RA ile75val polymorphism was significantly increased in *Alternaria*-sensitive moderate-severe asthmatics, 83% (0.627 allele frequency) compared to *Alternaria*-sensitive mild asthmatics, 57% (0.388 allele frequency). This was associated with increased sensitivity to IL-4 stimulation measured by significantly increased IL-4 stimulated CD23 expression on CD19+ and CD86+CD19+ B cells of *Alternaria*-sensitive moderate-severe asthmatics. IL-5 and IL-13 synthesis was significantly increased in *Alternaria*-sensitive moderate-severe asthmatics compared to mild asthmatics to *Alternaria *extract and Alt a1 stimulation. The frequency of HLA-DQB1*03 allele was significantly decreased in *Alternaria*-sensitive moderate-severe asthmatics compared to mild asthmatics, 39% versus 63%, with significantly decreased allele frequency, 0.220 versus 0.398.

**Summary:**

In children with *Alternaria*-sensitive moderate severe asthma, there was an increased Th2 response to *Alternaria *stimulation and increased sensitivity to IL-4 stimulation. This skewing towards a Th2 response was associated with an increased frequency of the IL-4RA ile75val polymorphism. In evaluating the HLA association, there was a decreased frequency of HLA-DQB1*03 in *Alternaria*-sensitive moderate severe asthmatic children consistent with previous studies suggest that HLA-DQB1*03 may be protective against the development of mold-sensitive severe asthma.

## Background

Asthma afflicts 6% to 8% of the United States population, and severe asthma represents approximately 10% of asthmatic patients [1]. This subset of severe asthmatic patients have significant morbidity and utilize health care resources disproportionately more compared to asthmatic patients with less severe disease. The current medication regimen of inhaled corticosteroids, leukotriene antagonists, and long-acting beta-2 agonists are usually inadequate to control severe asthma. Thus, it becomes important to understand the mechanism(s) as to why these patients have pulmonary inflammation that is not adequately controlled by current treatment regimens.

Several epidemiologic studies in the United States and Europe have linked *Alternaria *sensitivity to both persistence and severity of asthma [2-18]. *Alternaria alternata *spores are the most common airborne mold in the United States and are especially prevalent in the grain-growing areas of the Midwest. In addition, significant risk for acute asthma and life-threatening asthma has been associated with *Alternaria*-sensitive asthma when mold spore counts have been elevated [19-23]. Recently, Pasqualotto et al [24] coined the term severe asthma associated with fungal sensitization (SAFS) in adult patients with asthma in the United Kingdom. In their studies, sensitivity to *Aspergillus fumigatus *was the most prevalent (66%), followed by sensitivities to *Cladosporium *(52%) and to *Alternaria *(34%). Furthermore, treatment of these patients with itraconazole in a 32 week trial resulted in improved asthma quality of life (AQLQ), decreased IgE levels, and increased peak flow (PF).

The immunopathogenesis of atopic asthma is complex and multifunctional. Multiple genetic risk factors involving the inflammatory pathways, including polymorphisms of *IL-4RA*, *IL-4*, *IL-10*, *IL-13*, and *CD14*, have been described but are not present in the majority of patients. In particular, polymorphisms of *IL-4RA *and *IL-13 *have been associated with elevated IgE levels and asthma severity. We hypothesized that there are genotype similarities between *Alternaria*-sensitive moderate-severe asthma and allergic bronchopulmonary aspergillosis (ABPA). In our studies of ABPA, we identified genetic factors for the development of ABPA: (1) HLA-DR2 and HLA-DR5 restriction [25-27], and (2) *IL-4RA *single nucleotide polymorphism (SNP)[27,28]. Interestingly, the presence of HLA-DQ2 even in the presence of HLA-DR2/DR5 contributed to resistance of the development of ABPA. ABPA is a Th2 allergic hypersensitivity lung disease due to bronchial colonization of *A. fumigatus *that affects 1-2% of asthmatic and 7-9% of cystic fibrosis (CF) patients. Acute flares of ABPA are characterized by wheezing, pulmonary infiltrates, eosinophilia, increased levels of total IgE, and increased levels of anti-*A. fumigatus *specific IgE, IgG and IgA antibody levels. In the present study, we examined HLA class II antigens and IL-4RA polymorphisms in *Alternaria*-sensitive moderate severe asthmatic children.

## Methods

### Study Population and Sample Size

The study population consisted of both male and female Caucasian, African-American, Hispanic children 5 to 18 years old with mild, moderate, and severe persistent asthma recruited from the Allergy and Asthma clinics at Cardinal Glennon Children's Medical Center, Saint Louis University. Children were not stratified or excluded by race of gender. Classification of asthma severity was the GINA (NHBLI) criteria using day/night symptoms, pulmonary function, and medications. Patients were evaluated for allergen sensitivities by allergy prick skin testing (Multi-Test II; Lincoln Diagnostics, Decatur, IL) to *Alternaria alternata*, *Cladosporium herbarum*, *Helminthosporium sativum*, *Aspergillus fumigatus*, *Dermatophagoides pteronyssinus *and *farinae *(housedust mites, HDM), American/German cockroach, cat hair, dog epithelium, tree pollens (oak, hickory-pecan, maple/box elder, elm, ash, sycamore, walnut, juniper, birch), grass pollens (Johnson, Bermuda, June, timothy, bahia), and weed pollens (short and giant ragweed, plantain, sorrel, mugwort, hackberry, mulberry)(reagents obtained from Greer Laboratories, Lenoir, NC). Tests were regarded as positive when the mean diameter of the wheal was ≥ 3 mm. The study group consisted of *Alternaria*-sensitive moderate-severe asthma compared to *Alternaria*-sensitive mild asthmatics. The study was fully approved by the Saint Louis University Institutional Review Board (IRB #14611, approved 9-3-2008).

### *IL-4RA *genotyping by direct sequence analysis

*IL-4RA *polymorphisms were detected as previously described [27,28]. Genomic variants of *IL-4RA *were numbered on the basis of their location in *IL-4RA *mRNA sequence of gene bank accession number X52425. Five previously reported IL-4RA variants ile75val (rs1805010), glu400ala (rs1805011), cys431arg (rs1805012), ser503pro (rs1805015) and gln576arg (rs1801275)(numbering including the 25 amino acid signal peptide) were genotyped. Genomic variants in *IL-4RA *were identified by direct sequencing in both the forward and reverse direction. Both forward and reverse sequencing primers were used to maintain quality control. Primer sequences and conditions are available upon request. The presence of *IL-4RA *nucleotide polymorphisms was examined using the NCBI Blast program (http://ncbi.nlm.nih.gov/blast/bl2seq/wblast2; accession number 33833); homozygous/heterozygous SNPs were detected on the nucleotide chromatograph.

### *IL-13 *genotyping by PCR restriction fragment length polymorphism analysis

Genotyping was performed by PCR amplification of the genomic DNA region containing the arg110gln SNP (rs20541) followed by restriction digestion and comparison of size fragments to a standard size DNA ladder on gel electrophoresis, as previously described [27,28]. The expected product sizes are 236 bp for the wild type sequence and 178 bp for the arg110gln SNP. Complete digestion is confirmed by the presence of a 26 bp fragment from the *NLAIV *site in the primer and in the 5' end of the PCR product. Detailed PCR conditions are available upon request.

### IL-4 induction of B-cell CD23 expression

Peripheral blood mononuclear cells (PBMC) were isolated from venous blood by Ficoll-Hypaque density centrifugation as previously described [28]. PBMC were cultured at 1 × 10^6 ^cells/ml in 1 ml of RPMI 1640 supplemented with 10% FCS for 48 hours at 37°C in a 6% CO_2 _humidified atmosphere. The cultures were stimulated with rhuIL-4 (PeproTech, Inc) at 25 ng/ml. After 48 hours, the cells were washed and analyzed by flow cytometry.

### Flow cytometry

PBMC prior to culture and after culture were analyzed for induction of cell surface CD23 expression on B-cells, as previously described [27,28]. For cultures stimulated with IL-4, PBMC were washed and stained with murine monoclonal antibody to CD23-PE and CD20 Per-CP (Becton Dickinson). PBMCs were washed and fixed with 1% PBS buffered paraformaldehyde. Forward and side-scatter was performed to gate on the live lymphocyte population and further gated on CD20^+ ^cells for analysis using the CellQuest program (Becton Dickinson). A minimum of 10,000 cells were counted. Quantibrite PE flow cytometry beads (Becton Dickinson) were used to quantify the number of CD23 receptors per B-cell for each experiment. The beads contain a given number of PE molecules per bead. A linear equation was calculated from which the number of CD23 receptors per cell was extrapolated, and the total number of CD23 receptors expressed per B-cell determined.

### TH1/Th2 cytokines and chemokines

To determine Th1/Th2 *Alternaria*-specific T-cell response, *Alternaria *stimulated cultures were performed as previously described [27,28]. 1 × 10^6 ^PBL were cultured in 1 ml volume of RPMI supplemental with 10% FCS for 1 week in a humidified 5% CO_2 _atmosphere at 37°C. Cultures were stimulated in media alone, 25 mcg/ml of *Alternaria alternata *extract and 25 mcg/ml of Alt a1. The culture supernatant were obtained and frozen at -70°C until analyzed. *Alternaria *extract and Alt a1 were obtained from Dr. Hari Vijay. Measurement of Th1/Th2 culture supernatant cytokines and chemokines was performed by Flex Cytometric Bead Assay (BD Pharmingen) to measure IL-4, IL-5, IL-10, IL-13, IFN-γ, synthesis, as previously described [27,28].

### HLA typing

In order to examine HLA-DR and HLA-DQ allelic frequencies in *Alternaria*-sensitive asthmatic, HLA-DR and DQ typing was performed in the HLA Laboratory as previously described [25-27]. HLA-DRB1 alleles were detected by PCR amplification of genomic DNA with sequence specific primers (PCR-SSP; Dynal, Inc, Oslo, Norway). HLA-DQ typing were performed by PCR amplification of genomic DNA by using low resolution HLA-DQB allele specific primers identifying 5 HLA-DQ alleles (One Lambda, Canoga, Park, CA).

### Statistical analysis

The data for PFTs and cytokine levels were expressed as the mean ± SD and for IgE geometric mean ×/÷ SD. Statistical analysis using two-tailed Mann-Whitney U test was used comparing mold-sensitive moderate-severe asthma to other groups. Two-sided Fisher's exact test analysis was used comparing moderate-severe asthma to mild asthma. *P *values less than 0.05 were considered significant, using GraphPad InStat software package.

## Results

### Demographics

In Table 1, the demographics of *Alternaria*-sensitive moderate-severe asthma is compared to *Alternaria*-sensitive mild asthma in children. Comparison of *Alternaria*-sensitive moderate-severe asthmatics to *Alternaria*-sensitive mild asthmatics demonstrated that the groups were age and sex matched comparably. However, there were significantly greater percentage of African-Americans in the *Alternaria*-sensitive moderate-severe asthma group compared to the *Alternaria*-sensitive mild asthma group, 70% versus 36% (p = 0.0002). Medication use of omalizumab (p < 0.0001), high-dose and medium-dose inhaled corticosteroids (p < 0.0001 and p = < 0.0002, respectively), long-acting beta agonists (p < 0.0001) was significantly increased in *Alternaria*-sensitive moderate severe asthmatics compared to *Alternaria*-sensitive mild asthmatics. Immunotherapy was part of the treatment in 4% of *Alternaria*-sensitive moderate-severe asthmatics and 5% of *Alternaria*-sensitive mild asthmatics. The percentage of patients on immunotherapy is unlikely to affect the responses to *Alternaria *stimulation. Results of pulmonary function studies performed on current medications revealed that FVC, FEV-1, FEF-25-75, and FEV-1/FVC ratio were significantly decreased in *Alternaria*-sensitive moderate-severe asthma compared to *Alternaria*-sensitive mild asthma. Total serum IgE levels were significantly increased in *Alternaria*-sensitive moderate-severe asthma compared to *Alternaria*-sensitive mild asthma, 469 IU/ml versus 140 IU/ml (p < 0.0001). Children with *Alternaria*-sensitive moderate-severe asthma tended to have increased sensitivities to *Cladosporium *and *Aspergillus *as well. *Alternaria*-sensitive moderate-severe asthma had increased sensitivities to tree pollens (78% versus 57%, p = 0.01) and to weed pollens (68% versus 48%, p = 0.04).

**Table 1:** Demographics of children with *Alternaria*-sensitive moderate-severe asthma compared to *Alternaria*-sensitive mild asthma.

Study	Moderate-Severe (60)	Mild (49)	*P*
Age, years	11 ± 4	10 ± 3	
Sex, % male/female	62/38	64/36	
White/Black/Hispanic, %^#^	30/70/0	57/36/7	0.0002
Atopic dermatitis, %	33	26	
Medications, %^#^			
Omalizumab	28	0	<0.0001
ICS-H	36	4	<0.0001
ICS-M	52	18	0.0002
ICS-L	10	62	<0.0001
LABA	84	40	<0.0001
LTRA	77	64	
Immunotherapy, %	4	5	
Pulmonary function*			
FVC	88 ± 15	98 ± 11	<0.0001
FEV-1	78 ± 16	94 ± 11	<0.0001
FEF-25-75	64 ± 23	88 ± 23	<0.0001
FEV-1/FVC	85 ± 12	93 ± 8	<0.0001
IgE, IU/ml*	469 ×/÷ 3.51	140 ×/÷ 5.01	0.0001
Sensitivites, %^#^			
*Alternaria*	100	100	
*Cladosporium*	46	35	
*Helminthosporium*	32	28	
*Aspergillus*	43	28	
Der p and/or Der f	52	37	
Cat	46	28	
CR	28	22	
Trees	78	57	0.01
Grasses	56	54	
Weeds	68	48	0.04

Abbreviations: ICS-H, inhaled corticosteroid-high dose; ICS-M, -medium dose; ICS-L, -low dose; LABA, long-acting beta agonist; LTRA, leukotriene antagonist; IT, immunotherapy; FVC, forced vital capacity; FEV-1, forced expiratory volume 1 second; FEF, forced expiratory flow; CR, cockroach.

Pulmonary Function data expressed presented as Mean ± SD; IgE data expressed as Geometric Mean×/÷SD; Sensitivities data expressed as percentage of patients.

*P *value using Mann-Whitney U test* and Fisher's exact test^#^.

### IL-4RA and IL-13 polymorphisms

The results of IL-4RA single nucleotide polymorphisms (SNP) are seen in Table 2. The presence and allele frequency of IL-4RA ile75val SNP were significantly increased in *Alternaria*-sensitive moderate-severe asthmatics compared to *Alternaria*-sensitive mild asthmatics, 83% of patients versus 57% of patients (p = 0.005) and allele frequency 0.627 versus 0.388 (p = 0.012). This is similar to our studies in ABPA, where the frequency of IL-4RA ile75val was significantly increased compared to *Aspergillus*-sensitive asthmatics and CF patients. Other IL-4RA SNPS, glu400ala, ser503pro, and gln576arg tended to be increased frequency in *Alternaria*-sensitive asthmatics but were not statistically significant. However, the combination of 75val and 576arg, 75val576arg IL-4RA, was significantly increased in *Alternaria*-sensitive moderate-severe asthmatics, 63% versus 38% (p = 0.012). The IL-13 arg110gln SNP was similar in both moderate-severe and mild asthmatics, 31% versus 37%, with similar allele frequencies, 0.178 versus 0.204. The combination of the IL-4RA and IL-13 SNPs, 75val/576arg/110gln, was tended to be increased in *Alternaria*-sensitive moderate-severe asthmatics, 22% versus 8% (p = 0.07).

**Table 2:** IL-4RA and IL-13 polymorphisms in children with *Alternaria*-sensitive moderate-severe asthma compared to *Alternaria*-sensitive mild asthma.

Study	Moderate-Severe (60)	Mild (49)	*P*
IL-4RA SNPs			
ile75val	83 (0.627)	57 (0.388)	0.005 (0.012)
glu400ala	61 (0.390)	49 (0.265)	
cys431arg	15 (0.102)	22 (0.112)	
ser503pro	53 (0.347)	37 (0.214)	
gln576arg	75 (0.534)	59 (0.406)	
IL-13 SNP			
arg110gln	31 (0.178)	37 (0.204)	
75val/576arg	63	38	0.012
75val/110gln	31	17	
75val/576arg/110gln	22	8	0.07

Abbreviations: IL-4RA, IL-4 receptor alpha chain; SNP single nucleotide polymorphisms.

Data presented as percentage (%) of patients and in parentheses, allele frequency.

*P *value using Fisher's exact test.

### Up-regulation of CD23 expression

**Figure 1:**
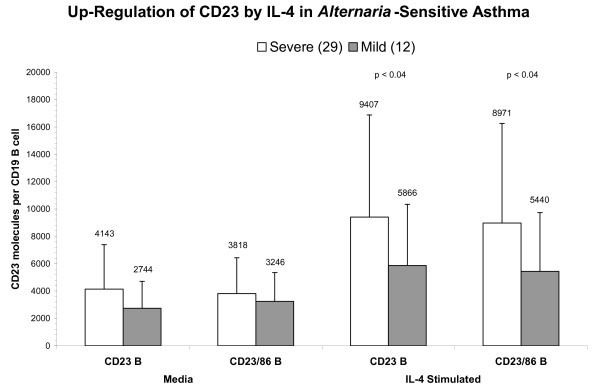
**Up-regulation of CD23 molecules by IL-4 stimulation on B cells**. Following IL-4 stimulation, *Alternaria*-sensitive moderate-severe asthmatics had a significantly increased CD23+ expression on both CD19+ and CD19+CD86+ B-cells compared to *Alternaria*-sensitive mild asthmatics (p < 0.04 and p < 0.04, respectively, Mann-Whitney U test). Data presented as Mean ± SD.

The up-regulation of CD23 molecules on B-cells by IL-4 stimulation is shown in Figure 1. In the absence of IL-4, the number of CD23 molecules decreased after 48 hours in media and was comparable in both *Alternaria*-sensitive moderate-severe and mild asthmatics. With IL-4 stimulation, the number of CD23 molecules per CD19+ and CD19+CD86+ B cell were significantly increased in *Alternaria*-sensitive moderate-severe asthmatics compared to *Alternaria*-sensitive mild asthmatics (p < 0.04 and p, 0.04, respectively).

### Cytokine synthesis

**Figure 2:**
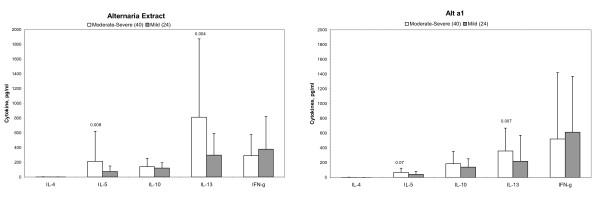
***Alternaria*-stimulated cytokine synthesis in *Alternaria*-sensitive moderate-severe asthma**. IL-5 and IL-13 synthesis was significantly increased in *Alternaria *extract stimulated T cells by *Alternaria*-sensitive moderate-severe asthmatic patients compared to *Alternaria*-sensitive mild asthma (p = 0.008 and p = 0.004, respectively) and to Alt a1 stimulated T cells by *Alternaria*-sensitive moderate-severe asthmatic patients compared to *Alternaria*-sensitive mild asthma (p = 0.07 and p = 0.007, respectively). In Asp f3 and Der p stimulated cultures, there were no significant differences of IL-5 and IL-13 synthesis comparing *Alternaria*-sensitive moderate-severe asthmatic versus *Alternaria*-sensitive mild asthmatics. Data presented as Mean ± SD. *P *value using Mann-Whitney U test.

In *Alternaria*-sensitive moderate-severe asthma, *Alternaria *extract stimulated lymphocytes had significantly increased synthesis of IL-5 and IL-13 compared to *Alternaria*-sensitive mild asthma (p = 0.008 and p = 0.004, respectively)(Figure 2). Similarly, IL-5 and IL-13 synthesis was increased to Alt a1 stimulated lymphocytes in *Alternaria*-sensitive moderate-severe asthmatics compared to *Alternaria*-sensitive mild asthmatics (p = 0.07 and p = 0.007, respectively). This would suggests that in *Alternaria*-sensitive moderate-severe asthma *Alternaria *exposure results in increased Th2 allergic inflammatory responses compared to *Alternaria*-sensitive mild asthma. Asp f1 and Der p1 stimulated IL-5 and IL-13 synthesis tended to be increased in *Alternaria*-sensitive moderate-severe asthmatics compared to *Alternaria*-sensitive asthmatics but was not significant (data not shown). This suggested that the increased Th2 cytokine synthesis was specific to *Alternaria *stimulation.

### HLA-DR and HLA-DQ typing

We subsequently examined frequencies of HLA-DR HLA-DP, and HLA-DQ in *Alternaria*-sensitive moderate-severe asthmatics (Table 3). The frequencies of HLA-DP were not significantly different comparing the groups (data not shown). The HLA-DQB1*03 allele was significantly decreased in *Alternaria*-sensitive moderate-severe asthmatics compared to *Alternaria *-sensitive mild asthmatics, 39% versus 63% (p = 0.02), with significantly decreased allele frequency, 0.220 versus 0.398 (p = 0.007). In previous studies, Chauhan et al (32) reported that HLA-DQB1*03 was present in 51% of 98 non-atopic controls, and in the dbMHC data base http://www.ncbi.nlm.nih.gov/projects/mhc/ihwg.cgi theHLA-DQB1*03 allele frequency was significantly increased in 78.5% of 1328 individuals in North America (p < 0.0001). Our results suggest a significant stratification compared to the background population frequencies. These preliminary results of decreased frequency of HLA-DQB1*03 in *Alternaria*-sensitive moderate-severe asthma in children is similar to that found in ABPA where HLA-DQB1*02 was decreased. It was determined that HLA-DQB1*02 was protective against the development of ABPA in *Aspergillus*-sensitive asthmatics and CF patients. These preliminary results of decreased frequency of HLA-DQB1*03 in *Alternaria*-sensitive moderate-severe asthmatics will need to be confirmed with a larger study population.

**Table 3:** HLA-DR and HLA-DQ allele frequencies in children with *Alternaria*-sensitive moderate-severe asthma compared to *Alternaria*-sensitive mild asthma.

Study	Moderate-Severe (60)	Mild (49)	*P*
HLA-DRB1			
*01	10 (0.051)	8 (0.041)	
*03	29 (0.153)	20 (0.102)	
*04	14 (0.076)	29 (0.153)	
*07	24 (0.127)	27 (0.143)	
*08	7 (0.034)	10 (0.061)	
*09	7 (0.034)	4 (0.020)	
*10	2 (0.008)	2 (0.010)	
*11	17 (0.093)	33 (0.184)	
*12	7 (0.034)	2 (0.010)	
*13	37 (0.195)	20 (0.112)	0.06
*14	3 (0.017)	2 (0.010)	
*15	27 (0.161)	27 (0.143)	
*16	3 (0.010)	2 (0.010)	
			
HLA-DQB1			
*02	42 (0.254)	33 (0184)	
*03	39 (0.220)	63 (0.398)	0.02 (0.007)
*04	12 (0.068)	8 (0.041)	
*05	31 (0.161)	20 (0.102)	
*06	44 (0.288)	51 (0.276)	

Data presented as percentage of patients with allele and in parentheses allele frequency.

*P *value using Fisher's exact test.

The allele frequency of HLA-DRB1*13 tended to be increased in *Alternaria*-sensitive moderate-severe asthma compared to *Alternaria*-sensitive mild asthma, 37% versus 20% (p = 0.06). The frequency of HLA-DRB1*13 in *Alternaria*-sensitive moderate-severe asthma was significantly increased compared to individuals in the dbMHC data base http://www.ncbi.nlm.nih.gov/projects/mhc/ihwg.cgi, HLA-DRB1*13 ranged from 0.5% of 1330 individuals in North America to 9.8% of 2587 individuals in Europe (p < 0.0001).

## Discussion

*Alternaria alternata *spores are the most common airborne mold in the United States and are especially prevalent in the grain-growing areas of the Midwest. Several epidemiologic studies in the United States and Europe have linked *Alternaria *sensitivity to both persistence and severity of asthma [2-18]. Many *Alternaria *allergens have been isolated and purified. Similar to *Aspergillus *allergens, these proteins have biologic activity on the respiratory epithelia in addition to inducing allergic inflammatory responses. Kauffman et al [29] reported that *Alternaria *and *Cladosporium *proteases had a direct effect on the bronchial epithelia causing pro-inflammatory cytokine synthesis and desquamation similar to *A. fumigatus *proteases; although *Aspergillus *proteases were more potent. In addition, Kheradmand et al [30] in a murine model demonstrated that *Alternaria *proteases promoted a chronic eosinophilic inflammation in the airways of mice exposed to these antigens. This is similar to the findings that Kurup's group identified in their murine model of allergic bronchopulmonary aspergillosis (ABPA). Another mechanism that may be operative in mold-induced asthma involves chitin, a major structural protein of the outer coating of fungi [31]. Chitin polarizes immune Th1 responses by suppressing Th2 responses. In humans, acidic mammalian chitinase degrades chitin shifting the responses toward a Th2 inflammatory response. Elevated chitinase has been associated with asthma and elevated IgE levels perhaps through an IL-13 pathway [32]. In the present studies, *Alternaria*-stimulated IL-5 and IL-13 synthesis was significantly increased in *Alternaria *-sensitive moderate-severe asthmatic children compared to *Alternaria*-sensitive mild asthmatics. Thus, increased Th2 responses to *Alternaria *in mold-sensitive moderate-severe asthmatic children appear to be important.

The immunopathogenesis of atopic asthma is complex and multifactorial. Allergic inflammation of the bronchial airways highlights the pathogenesis. Multiple genetic risk factors involving the inflammatory pathways, including polymorphisms of IL-4RA, IL-4, IL-10, IL-13, CD14, have been described but are not present in the majority of patients. We hypothesized that there are similarities of *Alternaria*-sensitive moderate-severe asthma and allergic bronchopulmonary aspergillosis (ABPA). In our studies of ABPA, we identified risk factors for the development of ABPA: (1) HLA-DR2 and HLA-DR5 restriction [25,26] and (2) IL-4RA single nucleotide polymorphism (SNP)[27,28].

Polymorphisms of the IL-4 receptor alpha chain (*IL-4RA*) and *IL-13 *have been associated with elevated IgE levels and asthma severity. There are eight naturally occurring single nucleotide polymorphisms (SNPs) of the *IL4RA *gene: ile75val, glu400ala, cys431arg, ser436leu, ser503pro, gln576arg, ser752ala, and ser786pro reported [33-38]. Studies have identified a number of these SNPs to be associated with atopy prevalence and asthma severity [33-38]. In the present study, IL-4RA ile75val was significantly increased in *Alternaria*-sensitive moderate-severe asthmatic children. Hershey et al [33] initially reported on a high prevalence of atopy and a gain-of-function in the IL-4R as measured by increased CD23 expression in patients with 576arg. This was also observed in the present study in children with *Alternaria*-sensitive moderate-severe asthma. Specifically, IL-4 stimulated CD23 up-regulation was observed on CD86+ B cells. CD86+ B cells are the subpopulation of B cells that secrete IgE, which correlates with the increased serum IgE seen in the patients with *Alternaria*-sensitive moderate-severe asthma. A subsequent study from Hershey's group found that the presence of these two variants (75val and 576arg) together resulted in elevated IL-4 dependent CD23 expression which was not observed when these SNPs were present alone [39]. Vladich et al (40) and Chen et al [41] reported that *IL-13 *arg110gln was associated with elevated IgE levels and increased severity of asthma [40,41]. This SNP has an allele frequency of approximately 20% in the Caucasian population. The *IL-13 *110gln polymorphism is significantly more active than the wild type IL-13 in stimulating STAT-6 phosphorylation, CD23 up-regulation, and IgE synthesis. Chen et al [41] also reported that combination of the *IL-4RA *SNPs, 75val and 576arg, and *IL-13 *SNP, 110gln, have been associated with atopy and asthma. This was observed in 22% of the children with *Alternaria*-sensitive moderate-severe asthma compared to 8% of children with mild asthma. In addition, Wenzel et al (1) reported that there was increased frequency of the ser503pro IL-4RA polymorphism in adults with severe asthma, which was not seen in this study.

In ABPA, we previously reported HLA-DR2 (HLA-DRB1*15 and B1*16)/DR5 (HLA-DRB1*11 and HLA-DRB1*12) restriction, and in particular HLA-DRB1*1501 and HLA-DRB1*1503 genotypes as a risk factor for the development of ABPA [25,26]. Interestingly, the presence of HLA-DQ2 even in the presence of HLA-DR2/DR5 contributed to resistance of the development of ABPA. In previous studies, we have identified HLA-DR restriction to *Alternaria *allergens in the development of *Alternaria*-sensitive moderate-severe asthma data not shown). In addition, HLA-DRB1*03 was significantly increased in mold sensitive moderate-severe asthmatic children compared to mold sensitive mild asthmatics. In *Alternaria*-sensitive moderate-severe asthmatic children the frequency of HLA-DRB1*03 trended to be increased but was not significant. However, HLA-DQB1*03 was significantly decreased in *Alternaria*-sensitive moderate-severe asthmatics. In previous studies, HLA-DQB1*03 was demonstrated to be associated with decreased *Alternaria *stimulated IL-5 and IL-13 synthesis. Thus, HLA-DQ3+ appears to be protective of development of *Alternaria*-sensitive severe asthma.

## Conclusions

In summary, we hypothesize that in children with *Alternaria*-sensitive moderate-severe asthma that there are genetic risk factors similar to those identified in ABPA. These include HLA-DR restriction, HLA-DQB1*03 protection, and IL-4RA polymorphisms. We propose that there is increased sensitivity to IL-4 and IL-13 mediated activities secondary to polymorphisms of *IL-4RA*. This is associated with HLA-DRB1*03 restriction and decreased HLA-DQB1*03 protection to *Alternaria *antigens that results in *Alternaria *stimulated skewing of *Alternaria *-specific Th2 cells, increased B-cell activity, and increased bronchial epithelial allergic inflammatory responses.

## Key words and Abbreviations

Asthma

Alternaria alternata

HLA class II antigens

Th2 cytokines

SNP: single nucleotide polymorphism; IL-4RA: interleukin 4 receptor alpha chain.

## Competing interests

The authors declare that they have no competing interests.

## Authors' contributions

APK conceived of the study and participated in its design and coordination.  HMV provided *Alternaria *extract and recombinant Alt a1.  BK performed cell cultures and PCR studies.  LAS performed HLA studies.  RG provided expertise in HLA studies.  JDW provided statistical support.  MRS provided technical expertise in PCR studies.  All authors read and approved the final manuscript.
